# Efficacy of Potentially Probiotic Fruit-Derived *Lactobacillus fermentum*, *L. paracasei* and *L. plantarum* to Remove Aflatoxin M_1_ In Vitro

**DOI:** 10.3390/toxins13010004

**Published:** 2020-12-23

**Authors:** Paloma Oliveira da Cruz, Clarisse Jales de Matos, Yuri Mangueira Nascimento, Josean Fechine Tavares, Evandro Leite de Souza, Hemerson Iury Ferreira Magalhães

**Affiliations:** 1 Laboratory of Toxicology, Department of Pharmaceutical Sciences, Health Sciences Center, Federal University of Paraíba, João Pessoa 58051-900, Brazil; paloma.oliveira05@hotmail.com (P.O.d.C.); clarissejmatos@hotmail.com (C.J.d.M.); hemersonufpb@gmail.com (H.I.F.M.); 2 Unity for Characterization and Analysis, Institute for Research in Pharmaceuticals and Medications, Federal University of Paraíba, João Pessoa 58051-900, Brazil; yurimangueira@ltf.ufpb.br (Y.M.N.); josean@ltf.ufpb.br (J.F.T.); 3 Laboratory of Food Microbiology, Department of Nutrition, Health Sciences Center, Federal University of Paraíba, João Pessoa 58051-900, Brazil

**Keywords:** aflatoxin M_1_, detoxification, *Lactobacillus*, probiotics, binding

## Abstract

This study evaluated the efficacy of potentially probiotic fruit-derived *Lactobacillus* isolates, namely, *L. paracasei* 108, *L. plantarum* 49, and *L. fermentum* 111, to remove aflatoxin M_1_ (AFM_1_) from a phosphate buffer solution (PBS; spiked with 0.15 µg/mL AFM_1_). The efficacy of examined isolates (approximately 10^9^ cfu/mL) as viable and non-viable cells (heat-killed; 100 °C, 1 h) to remove AFM_1_ was measured after 1 and 24 h at 37 °C. The recovery of AFM_1_ bound to bacterial cells after washing with PBS was also evaluated. Levels of AFM_1_ in PBS were measured with high-performance liquid chromatography. Viable and non-viable cells of all examined isolates were capable of removing AFM_1_ in PBS with removal percentage values in the range of 73.9–80.0% and 72.9–78.7%, respectively. Viable and non-viable cells of all examined *Lactobacillus* isolates had similar abilities to remove AFM_1_. Only *L. paracasei* 108 showed higher values of AFM_1_ removal after 24 h for both viable and non-viable cells. Percentage values of recovered AFM_1_ from viable and non-viable cells after washing were in the range of 13.4–60.6% and 10.9–47.9%, respectively. *L. plantarum* 49 showed the highest AFM_1_ retention capacity after washing. *L. paracasei* 108, *L. plantarum* 49, and *L. fermentum* 111 could have potential application to reduce AFM_1_ to safe levels in foods and feeds. The cell viability of examined isolates was not a pre-requisite for their capacity to remove and retain AFM_1_.

## 1. Introduction

Aflatoxins are fungal secondary metabolites toxic to humans and animals, causing carcinogenic, mutagenic, teratogenic, and immunosuppressive effects [1]. Aflatoxins are produced by toxigenic *Aspergillus flavus*, *A. parasiticus*, and *A. nomius* isolates growing in a variety of food and feed commodities [2]. These metabolites are very stable to autoclaving, pasteurization, and other food processing procedures [3].

Aflatoxin M_1_ (AFM_1_) is a 4-hydroxy derivative of aflatoxin B_1_ (AFB_1_), which, although approximately ten-fold less toxigenic than aflatoxin B_1_, exerts cytotoxic, genotoxic, and carcinogenic effects in a variety of species [2], being classified as belonging to group 1 (i.e., carcinogenic to humans) by the International Agency for Cancer Research [4]. AFM_1_ is formed in the liver and excreted through the milk of lactating animals that have consumed feed contaminated with AFB_1_. Approximately 0.3–6.2% of AFB_1_ ingested by livestock is converted to AFM_1_ in milk [5]. In Brazil and the USA, the maximum allowable limit of AFM_1_ in raw milk is 0.5 µg/L [6,7]. The European Union has set a maximum limit of AFM_1_ of 0.05 µg/L for raw milk, heat-treated milk, and milk used in dairy products formulation [8].

Control of aflatoxin in food and feed can be primarily achieved by a prevention of mold contamination and growth with the adoption of improved agricultural practices and control of storage conditions, as well as by the detoxification of contaminated products through chemical (e.g., ammonia, hydrogen peroxide, alkalis, and acids) or physical methods (e.g., heat, radiations, ultraviolet, and microwave) [9]. Some methods used for aflatoxins decontamination, although they have been shown to be effective to a certain extent, may have some drawbacks, such as negative impacts on nutritional and sensory characteristics of foods, production of potentially toxic by-products, or non-suitability for use in solid foods [2,9].

Use of lactic acid bacteria (LAB) has been considered a safe and environmentally friendly biological method for the detoxification of aflatoxins in foods and feeds [10,11]. Studies have found a variable capability among probiotic *Lactobacillus* species or isolates to bind aflatoxins [12,13,14]. These studies have mostly used commercial *Lactobacillus* cultures or isolates from dairy origin. Although a number of *Lactobacillus* isolates recovered from fruit, vegetables, or their processing by-products have shown good performance in in vitro tests for the selection of probiotics [15,16,17], none of these isolates have been examined for their capacity to remove aflatoxins. The use of select probiotic *Lactobacillus* isolates has been considered a promising biological tool for removing aflatoxins from foods through adsorption when compared to chemical and physical treatments. Furthermore, although still the fastest method for retaining high detoxification efficacy [18,19], many chemical agents are nonedible materials and need to be eliminated after aflatoxin decontamination [20,21], while *Lactobacillus* species have been usually considered safe for use in foods [16,17].

Considering the available evidence, it was expected that fruit-derived *L. fermentum*, *L. paracasei*, and *L. plantarum* isolates with aptitudes to be used as probiotics would be able to remove AFM_1_ in a prospective view for application in food and feed detoxification. To test this hypothesis, this study evaluated the efficacy of these isolates as viable and non-viable (heat-killed) cells, in the removal of AFM_1_ in vitro, as well as the recovery of the AFM_1_ bound to bacterial cells.

## 2. Results and Discussion

**Figure 1:**
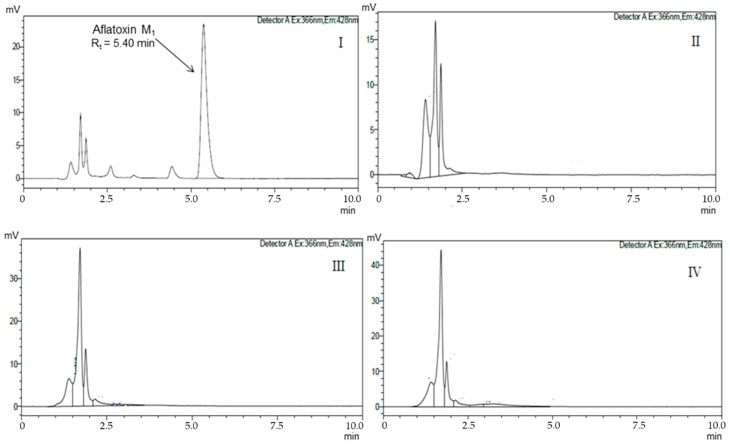
Chromatograms of aflatoxin M_1_ (AFM_1_) quantification in positive and negative control. (**I**) Positive control: phosphate buffer solution (PBS) with AFM_1_. R_t_ = Retention time of AFM_1_ in phosphate buffer solution; chromatographic peak area corresponding to AFM_1_; (**II**) Negative control after 1 h of incubation: PBS + *L. paracasei* 108; (**III**) Negative control after 1 h of incubation: PBS + *L. plantarum* 49; (**IV**) Negative control after 1 h of incubation: PBS + *L. fermentum* 111.

**Figure 2:**
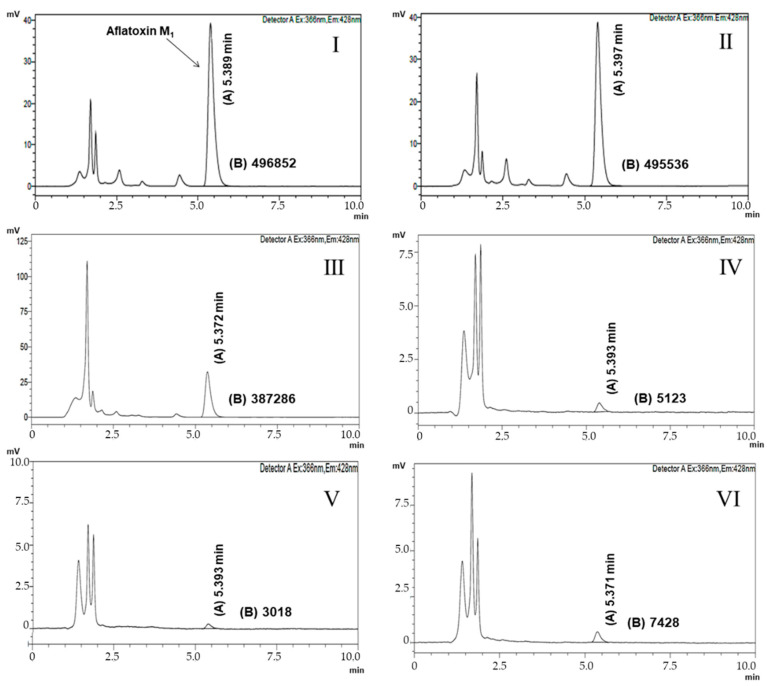
Chromatograms of aflatoxin M_1_ (AFM_1_) quantification in PBS. (**I**) Chromatogram of assays after 1 h of incubation: PBS + AFM_1_ + *L. paracasei* 108; (**II**) Chromatogram of assays after 1 h of incubation: PBS + AFM_1_ + *L. plantarum* 49; (**III**) Chromatogram of assays after 1 h of incubation: PBS + AFM_1_ + *L. fermentum* 111; (**IV**) AFM_1_ recovery chromatogram of *L. paracasei* 108 and AFM_1_ complex after 1 h of incubation; (**V**) AFM_1_ recovery chromatogram of *L. plantarum* 49 and AFM_1_ complex after 1 h of incubation; (**VI**) AFM_1_ recovery chromatogram of *L. fermentum* 111 and AFM_1_ complex after 1 h of incubation. (**A**) Retention time (min) of aflatoxin M_1_ in phosphate buffer solution; (**B**) chromatographic peak area corresponding to aflatoxin M_1_.

Chromatograms for the quantification of AFM_1_ in positive control, negative control, as well as in samples with viable cells of *L. paracasei* 108, *L. plantarum* 49, and *L. fermentum* 111 are shown in Figure 1. Chromatograms for the quantification of AFM_1_ in assays evaluating the recovery of AFM_1_ from cells after 1 h of incubation are shown in Figure 2.

Results of the capability of viable and heat-killed (non-viable) cells of *L. paracasei* 108, *L. plantarum* 49, and *L. fermentum* 111 for removing AFM_1_ in PBS are presented in Table 1. Viable and heat-killed cells of all examined *Lactobacillus* isolates were able to remove AFM_1_ in PBS, with removal percentage values in the range of 73.0 ± 1.2–80.0 ± 1.7% and 72.9 ± 1.1–78.7 ± 1.2%, respectively. Viable and heat-killed cells of the three examined isolates had similar values (*p* > 0.05) of AFM_1_ removal. Only *L. paracasei* 108 had higher values (*p* ≤ 0.05) of AFM_1_ removal after 24 h for both viable and heat-killed cells compared to 1 h. Higher values of AFM_1_ removal (*p* ≤ 0.05) after 1 h were found for *L. plantarum* 49 and *L. fermentum* 111, but the three examined isolates had similar values of AFM_1_ removal (*p* > 0.05) after 24 h.

Previous studies have also verified that the capacity of LAB—either as viable or non-viable cells, of binding aflatoxins (e.g., aflatoxin B1, ochratoxin, trichothecene, and AFM_1_) in PBS, laboratory media, or dairy matrices (e.g., milk and yoghurt)—varies in an isolate-dependent manner [2,11,22,23]. Aflatoxins bind to the surface components of LAB cells and variations in aflatoxin’s binding capacities among LAB species or isolates could be associated with differences in the bacterial cell wall and cell envelope structures [7]. Early investigations have found lower capacity of AFM_1_ removal by viable and/or heat-killed cells of different LAB (e.g., *L. plantarum*, *L. acidophilus*, *L. reuteri*, *L. johnsonii*, *L. rhamnosus*, *L. bulgaricus*, and *Streptococcus thermophilus*) [2,22,23], including probiotic *L. casei* [10], compared to *L. paracasei* 108, *L. plantarum* 49, and *L. fermentum* 111. The efficacy of AFM_1_ removal from PBS as high (>60%) as those found for *Lactobacillus* isolates examined in this study was reported to *L. plantarum* MON03 and *L. rhamnosus* GAF01 after 6 or 24 h of incubation [24].

Results of the AFM_1_ retention capacity of the viable and heat-killed cells of *L. paracasei* 108, *L. plantarum* 49, and *L. fermentum* 111 after washing with PBS are presented in Table 2. Percentage values of recovered AFM_1_ from viable and heat-killed cells were in the range of 13.4 ± 1.5–60.6 ± 1.6% and 10.9 ± 1.2%–47.9 ± 1.5%, respectively. The highest values of recovered AFM_1_ after 1 and 24 h were found for *L. fermentum* 111 and *L. paracasei* 108, respectively, for both viable and heat-killed cells. Only for *L. fermentum* 111 did the values of recovered AFM_1_ decrease after 24 h for viable and heat-killed cells; for *L. paracasei* 108 and *L. plantarum* 49, these values varied with the viability/non-viability of cells and incubation time period. Overall, *L. plantarum* 49 had the higher AFM_1_ retention capacity after washing. Variations in aflatoxin release have been linked to the differences in binding sites in different LAB isolates, or even in these binding sites being very similar. They could have minimal differences depending on each isolate [13,25,26].

For all examined isolates, the values of recovered AFM_1_ decreased after 24 h of incubation, indicating that AFM_1_ retention capacity increased when the length of the contact time increased. There was no clear association between the capability of removing AFM_1_, initially, and of retaining AFM_1_ after washing among examined isolates. Interestingly, a study with different *Lactobacillus* species found lower AFM_1_ removal values than those found in this study, although the recovery of AFM_1_ from bacterial cells was lower in the former [11].

Heat treatment positively affected the capability of retaining AFM_1_ in *L. paracasei* 108 after 1 h of incubation, as well as of *L. plantarum* 49 and *L. fermentum* 111 after 24 h of incubation. Heating could increase the interaction capacity of bacterial cells/aflatoxin complexes by causing an increased exposure of the cell wall components, primarily polysaccharides and peptidoglycans, which act as binding sites to aflatoxin [14]. However, the destruction of specific components of the bacterial cell wall by heating, causing the denaturation of proteins and increased cell surface hydrophobicity, has been cited to result in a decreased capability of LAB cells of binding AFM_1_ [7]. An increased capability of removing aflatoxin B1 was also found in *L. rhamnosus* after heating [27].

The recovery of the AFM_1_ bound to the cells of examined *Lactobacillus* isolates after washing indicates that the binding was not strong and could not involve a non-covalent weak bond, but probably a physical association of AFM_1_ with hydrophobic sites in the bacterial cell wall [13,20,25]. The lower AFM_1_ recovery values found for the examined isolates could be linked to the interaction of AFM_1_ molecules retained in the bacterial cell wall with other AFM_1_ molecules retained in adjacent cells, forming a type of cross-linked matrix that avoids aflatoxin release during washing [10]. Probably, the efficacy of this type of cross-linked matrix decreased over time for *L. paracasei* 108 and *L. plantarum* 49. Although some authors have reported that a part of non-recovered AFM_1_ might be degraded or biotransformed by a *Lactobacillus* metabolism [2,7], most of the available literature has indicated that aflatoxins are not removed by the metabolism of LAB, but because of a physical bound to the molecular components of bacterial cells, primarily peptidoglycans from the cell wall [19,21,25].

In agreement with available literature, the results of this study showed that the cell viability of the examined isolates is not a prerequisite for the removal and retaining of AFM_1_ [13,28]. Cell concentration as high as 10^8^–10^9^ CFU/mL of viable or non-viable LAB is typically needed to reach a level of aflatoxins removal of ≥ 50% [22,28].

## 3. Conclusions

Results showed that potentially probiotic *L. fermentum* 111, *L. paracasei* 108, and *L. plantarum* 49 isolated from fruit processing by-products are capable of binding AFM_1_ in vitro when assayed as either viable or non-viable cells. The recovery of AFM_1_ from bacterial cell complexes varied with the examined isolate and contact time. Non-viable cells had a higher capability for retaining AFM_1_ after 1 or 24 h of incubation. These results indicate that *Lactobacillus* isolates recovered from fruit with performance compatible to use as probiotics could have a satisfactory aflatoxin binding capacity, which could be exploited as a biological tool for the detoxification of foods and feeds, particularly, for the removal and restoration of AFM_1_ to safe levels. Further studies are needed to investigate the mechanisms involved in removal of AFM_1_ by these isolates and possible factors affecting the stability of formed complexes, including when exposed to conditions mimicking the human gastrointestinal tract.

## 4. Materials and Methods

### 4.1. Chemicals, Bacterial Isolates, and Inoculum Preparation

The AFM_1_ standard was obtained from Sigma Aldrich (St. Louis, MO, USA). High-performance liquid chromatography (HPLC) grade solvents were obtained from Merck (Darmstadt, Germany).

The isolates *Lactobacillus plantarum* 49, *L. fermentum* 111, and *L. paracasei* 108 were examined separately for the removal of AFM_1_. These isolates were recovered from fruit processing by-products, identified with a partial 16S rRNA gene sequence analysis and characterized as potential candidates for use as probiotics [17]. Stocks were stored at −20 °C in de Man, Rogosa, and Sharpe (MRS) broth (HiMedia, Mumbai, India) with glycerol (20 mL/100 mL; Sigma-Aldrich, St. Louis, MO, USA). Working cultures were maintained aerobically on MRS agar (HiMedia, Mumbai, India) at 4 °C and transferred to a new media monthly. Prior to use in assays, each isolate was cultivated anaerobically (Anaerobic System Anaerogen, Oxoid, Hampshire, UK) in MRS broth at 37 °C for 20–24 h (to reach the stationary growth phase), harvested by centrifugation (4500× *g*, 15 min, 4 °C), washed twice, and resuspended in phosphate buffer solution (PBS; 50 mM K_2_HPO_4_/KH_2_PO_4_; pH 6.9) to obtain cell suspensions with an optical density reading at 660 nm (OD_660_) of 0.5. This suspension had viable counts of approximately 1.1 × 10^9^ CFU/mL for each isolate when plated in MRS agar.

### 4.2. Evaluation of AFM_1_ Removal and Recovery of AFM_1_ from Bacterial Cells

The capability of examined *Lactobacillus* isolates to remove AFM_1_ in PBS was assessed with viable and non-viable bacterial cell suspensions. To obtain non-viable bacterial cells, *Lactobacillus* cell suspensions were inactivated by boiling at 100 °C for 1 h. No visible colonies were found when heat-treated cell suspensions (named heat-killed cells) were plated onto MRS agar and followed by anaerobic incubation (using Anaerobic System Anaerogen, Oxoid, Hampshire, UK) for 48 h. For testing the AFM_1_ removal capability, 1 mL of test isolate suspension (pure culture of viable and heat-killed cells) was mixed with 1.5 mL of PBS, previously spiked with 0.15 µg/mL AFM_1_, and incubated aerobically at 37 °C [28]. After 1 and 24 h of incubation, the mixture was centrifuged (1500× *g*, 15 min, 4 °C) and the AFM_1_ content in the supernatant was determined by HPLC, as detailed in Section 4.3.

Cell pellets collected from each monitored incubation period (contact time) were evaluated for the recovery of AFM_1_ from cell complexes. Obtained pellets were washed with 1.5 mL of fresh PBS, the cells were re-pelleted (1500× *g*, 15 min, 4 °C), and supernatant was collected for the quantification of released AFM_1_ [18]. For each isolate, a positive control consisting of free cells suspended in PBS with 0.15 µg/mL AFM_1_, and a negative control, consisting of bacterial cells (viable or heat-killed), suspended in PBS were used.

### 4.3. Quantification of AFM_1_

The quantification of AFM_1_ in supernatants was done with high-performance liquid chromatography (HPLC) using a Shimadzu (Prominense, Tokyo, Japan) HPLC system, equipped with an auto sampler SIL 20A HT (Prominense, Shimadzu, Tokyo, Japan), fluorescence detector RF-20A (Prominense, Shimadzu, Tokyo, Japan), an LC-20AT pump (Prominense, Shimadzu, Tokyo, Japan), oven CTO-20A (Prominense, Shimadzu, Tóquio, Japão), a CBM-20A controller (Prominense, Shimadzu, Tokyo, Japan), a CLC-ODS (M) reverse phase column (4.6 × 150 mm; Shim-Pack, Prominense, Shimadzu, Tokyo, Japan) and pre-column G-ODS-4 (1.0 × 4.0 mm; Shim-Pack, Prominense, Shimadzu, Tokyo, Japan).

Chromatographic conditions were the same as those described in a previous study [7]. Excitation and emission wavelengths were 366 and 428 nm, and the injection volume was 20 μL. The mobile phase was water:methanol:acetonitrile (6:2:2) and the flow rate was 1 mL/min. The calibration curve was constructed using six concentrations of AFM_1_ standard diluted in acetonitrile (20–60 ng/mL), performed in triplicate. From this analysis, the equation *y* = 2E+07*x* + 873,267 (*r*^2^ > 0.99) was obtained. The limit of detection (LOD) and limit of quantification (LOQ) were estimated based on Resolution n° 899 of the Brazilian Agency for Health Surveillance [29]. The LOD and LOQ of AFM_1_ were 0.20 and 0.67 ng/mL, respectively.

The percentage of AFM_1_ removed by each isolate was determined with the Equation (1) [22,27,30]:

100 × [1 − (peak area of chromatographic peak of sample)/area of positive control chromatographic peak)].(1)

### 4.4. Statistical Analysis

Assays were done in triplicate in three independent experiments (repetitions). A Kolmogorov–Smirnov normality test was run to assess whether obtained results had normal distribution. Results (average data ± standard deviation) were submitted to a one-way analysis of variance (ANOVA), followed by Tukey’s test, considering a *p* value of ≤ 0.05 for significance. Statistical analyses were done with IBM SPSS Statistics 20 (Armonk, NY, USA).

## Figures and Tables

**Table 1:** Percentage (average values ± standard deviation) of aflatoxin M_1_ (AFM_1_) removal in phosphate buffer solution by *L. paracasei* 108, *L. plantarum* 49, and *L. fermentum* 111.

Isolates	AFM_1_ Removal (%)			
	1 h-Incubation		24 h-Incubation	
	Viable Cells	Heat-Killed Cells	Viable Cells	Heat-Killed Cells
*L. paracasei* 108	73.0 ± 1.2 ^b^^,B^	72.9 ± 1.1 ^b,B^	78.9 ± 0.5 ^a,A^	78.7 ± 1.2 ^a,A^
*L. plantarum* 49	78.1 ± 1.6 ^a,A^	75.8 ± 1.0 ^a,A,B^	77.0 ± 2.7 ^a,A^	76.6 ± 1.5 ^a,A^
*L. fermentum* 111	78.6 ± 2.1 ^a,A^	78.4 ± 0.65 ^a,A^	80.0 ± 1.7 ^a,A^	78.3 ± 2.5 ^a,A^

Different small letters in the same row (a,b) denote a significant difference (*p* ≤ 0.05) among values, based on Tukey’s test; different capital letters in the same column (A,B) denote a significant difference among values (*p* ≤ 0.05), based on Tukey’s test.

**Table 2:** Percentage (average values ± standard deviation) of recovered aflatoxin M_1_ (AFM_1_) in solution after washing with phosphate buffer solution.

Isolates	AFM_1_ Recovery, %			
	1 h-Incubation		24 h-Incubation	
	Viable Cells	Heat-Killed Cells	Viable Cells	Heat-Killed Cells
*L. paracasei* 108	34.6 ± 1.1 ^b,B^	28.5 ± 1.7 ^d,C^	31.7 ± 1.2 ^c,A^	40.3 ± 1.6 ^a,A^
*L. plantarum* 49	13.4 ± 1.5 ^c,C^	43.8 ± 1.5 ^a,B^	18.8 ± 1.0 ^b,B^	10.9 ± 1.2 ^d,C^
*L. fermentum* 111	60.6 ± 1.6 ^a,A^	47.9 ± 1.5 ^b,A^	14.1 ± 1.4 ^c,C^	14.9 ± 1.6 ^c,B^

Different small letters in the same row (a–c) denote a significant difference (*p* ≤ 0.05) among values, based on Tukey’s test; different capital letters in the same column (A,B) denote a significant difference among values (*p* ≤ 0.05), based on Tukey’s test.

## References

[1] Bhat R., Rai R.V., Karim A.A. (2010). Mycotoxins in food and feed: Present status and future concerns. Compr. Rev. Food Sci. Food Saf..

[2] Elsanhoty R.M., Salam S.A., Ramadan M.F., Badr F.H. (2014). Detoxification of aflatoxin M1 in yoghurt using probiotics and lactic acid bacteria. Food Cont..

[3] Koppen R., Koch M., Slegel D. (2010). Determination of mycotoxins in foods: Current state of analytical methods and limitations. Appl. Microbiol. Biotechnol..

[4] International Agency for Research on Cancer (2002). Some traditional herbal medicine, some mycotoxins and styrene. Monographs on the Evaluation of Carginogenic Risks to Humans.

[5] Ayar A., Sert D., Lon A.H. (2007). A study on the occurrence of aflatoxin in raw milk due to feeds. J. Food Saf..

[6] Ministry of Health, National Agency for Health Surveillance, Brazilian Legislation (2011). On the Maximum Tolerated Limit (MTL) for Mycotoxins in Foods.

[7] Corassin C.H., Bovo F., Rossim R.E., Oliveira C.A.F. (2013). Efficiency of *Saccharomyces cerevisiae* and lactic acid bacteria strains to bind aflatoxin M1 in UHT skim milk. Food Cont..

[8] European Commission (2006). Commission Regulation (EC) No 401/2006 of 23 February 2006 laying down the methods of sampling and analysis for the official control of the levels of mycotoxins in foodstuffs. Off. J. Eur. Union.

[9] Wang B., Mahoney N.E., Pan Z., Khir R., Wu B., Ma H., Zhao L. (2016). Effectiveness of pulsed light treatment for degradation and detoxification of aflatoxin B1 and B2 in rough rice and rice bran. Food Cont..

[10] Hernandez-Mendoza A., Garcia H.S., Steele J.L. (2009). Screening of *Lactobacillus casei* strains for their ability to bind aflatoxin B1. Food Chem. Toxicol..

[11] Serrano-Niño J.C., Cavazos-Garduño A., Hernandez-Mendoza A., Applegate B., Ferruzzi M.C., Martin-González M.F.S., García H.S. (2013). Assessment of probiotic strains ability to reduce the bioaccessibility of aflatoxin M1 in artificially contaminated milk using an in vitro digestive model. Food Cont..

[12] Onilude A.A., Fagade O.E., Bello M.M., Fadahunsi I.F. (2005). Inhibition of aflatoxin-producing aspergilli by lactic acid bacteria isolates from indigenously fermented cereal gruels. Afr. J. Biotechnol..

[13] Azeem N., Nawaz M., Anjum A.A., Saeed S., Sana S., Mustafa A., Yousuf M.R. (2019). Activity and anti-aflatoxigenic effect of indigenously characterized probiotic lactobacilli against *Aspergillus flavus*—A common poultry feed contaminant. Animals.

[14] Elsanhoty R.M., Ramadan M.F., El-Gohery S.S., Abol-Ela M.A.A. (2013). Ability of selected microorganisms for removing aflatoxins in vitro and fate of aflatoxins in contaminated wheat during baladi bread baking. Food Cont..

[15] Naeem M., Ilyas M., Haider S., Baig S., Saleem M. (2012). Isolation characterization and identification of lactic acid bacteria from fruit juices and their efficacy against antibiotics. Pak. J. Bot..

[16] Ilha E.C., Silva T., Lorenz J.G., De Oliveira Rocha G., Sant’Anna E.S. (2015). *Lactobacillus paracasei* isolated from grape sourdough: Acid, bile, salt, and heat tolerance after spray drying with skim milk and cheese whey. Eur. Food Res. Technol..

[17] Garcia E.F., Luciano W.A., Xavier D.E., Da Costa W.C., De Sousa Oliveira K., Franco O.L., De Morais M.A.J., Lucena B.T., Picão R.C., Magnani M. (2016). Identification of lactic acid bacteria in fruit pulp processing byproducts and potential probiotic properties of selected *Lactobacillus* strains. Front. Microbiol..

[18] Ahlberg S.H., Joutsjoki V., Korhonen H.J. (2015). Potential of lactic acid bacteria in aflatoxin risk mitigation. Int. J. Food Microbiol..

[19] Khanian M., Karimi-Torshizi M.A., Allameh A. (2019). Alleviation of aflatoxin-related oxidative damage to liver and improvement of growth performance in broiler chickens consumed *Lactobacillus plantarum* 299v for entire growth period. Toxicon.

[20] Assaf J.C., Nahle S., Chokr A., Louka N., Atoui A., El Khoury A. (2019). Assorted methods for decontamination of aflatoxin m1 in milk using microbial adsorbents. Toxins.

[21] Kim S., Lee H., Lee S., Lee J., Ha J., Choi Y., Yoon Y., Choi K.H. (2017). Microbe-mediated aflatoxin decontamination of dairy products and feeds. J. Dairy Sci..

[22] El-Nezami H., Kankaanpää P., Salminen S., Ahokas J. (1998). Physicochemical alterations enhance the ability of dairy strains of lactic acid bacteria to remove aflatoxin from contaminated media. J. Food Prot..

[23] Pierides H., El-Nezami K., Peltonem S., Salminem J., Ahokas J.T. (2000). Ability of dairy strains of lactic acid bacteria to bind aflatoxin M1 in a food model. J. Food Prot..

[24] Jebali R., Abbes S., Salah-Abbès J.B., Younes R.B., Haous Z., Oueslati R. (2014). Ability of *Lactobacillus plantarum* MON03 to mitigate aflatoxins (B1 and M1) immunotoxicities in mice. J. Immunotoxicol..

[25] Panwar R., Kumar N., Kashyap V., Ram C., Kapila R. (2019). Aflatoxin M_1_ detoxification ability of probiotic lactobacilli of Indian origin in in vitro digestion model. Prob. Antimicrob. Proteins.

[26] Risa A., Divinyi D.M., Baka E., Krifaton C. (2017). Aflatoxin B1 detoxification by cell-free extracts of *Rhodococcus* strains. Acta Microbiol. et Immunol. Hung..

[27] Abbès S., Salah-Abbès J.B., Sharafi H., Jebali R., Noghabi K.A., Oueslati R. (2013). Ability of *Lactobacillus rhamnosus* GAF01 to remove AFM1 in vitro and to counteract AFM1 immunotoxicity in vivo. J. Immunotoxicol..

[28] Bovo F., Corassin C.H., Rosim R.E., De Oliveira C.A. (2012). Efficiency of lactic acid bacteria strains for decontamination of aflatoxin M1 in phosphate buffer saline solution and in skim milk. Food Bioproc. Technol..

[29] Brazilian Legislation, Ministry of Health, National Agency for Health Surveillance (2003). Guide for Validation of Analytical and Bioanalytical Methods. National Agency for Health Surveillance. Resolution nº899.

[30] Haskard C.A., El-Nezami H.S., Kankaanpää P.E., Salminen S., Ahokas J.T. (2001). Surface binding of aflatoxin B1 by lactic acid bacteria. Appl. Environ. Microbiol..

